# Precisely Tracking Childhood Death

**DOI:** 10.4269/ajtmh.16-0302

**Published:** 2017-06-05

**Authors:** Tamer H. Farag, Jeffrey P. Koplan, Robert F. Breiman, Shabir A. Madhi, Penny M. Heaton, Trevor Mundel, Jaume Ordi, Quique Bassat, Clara Menendez, Scott F. Dowell

**Affiliations:** 1 Bill & Melinda Gates Foundation, Seattle, Washington;; 2 Emory Global Health Institute, Atlanta, Georgia;; 3 International Association of National Public Health Institutes, Atlanta, Georgia;; 4 National Institute for Communicable Diseases, National Health Laboratory Service, Johannesburg, South Africa;; 5 ISGlobal, Barcelona Centre for International Health Research (CRESIB), Hospital Clínic, Universitat de Barcelona, Barcelona, Spain;; 6 Department of Pathology, Hospital Clinic, Universitat de Barcelona, Barcelona, Spain;; 7 Centro de Investigação em Saúde de Manhiça (CISM), Maputo, Mozambique;; 8 Institució Catalana de Recerca i Estudis Avançats (ICREA), Barcelona, Spain

## Abstract

Little is known about the specific causes of neonatal and under-five childhood death in high-mortality geographic regions due to a lack of primary data and dependence on inaccurate tools, such as verbal autopsy. To meet the ambitious new Sustainable Development Goal 3.2 to eliminate preventable child mortality in every country, better approaches are needed to precisely determine specific causes of death so that prevention and treatment interventions can be strengthened and focused. Minimally invasive tissue sampling (MITS) is a technique that uses needle-based postmortem sampling, followed by advanced histopathology and microbiology to definitely determine cause of death. The Bill & Melinda Gates Foundation is supporting a new surveillance system called the Child Health and Mortality Prevention Surveillance network, which will determine cause of death using MITS in combination with other information, and yield cause-specific population-based mortality rates, eventually in up to 12–15 sites in sub-Saharan Africa and south Asia. However, the Gates Foundation funding alone is not enough. We call on governments, other funders, and international stakeholders to expand the use of pathology-based cause of death determination to provide the information needed to end preventable childhood mortality.

Although global childhood mortality has dropped by half since 1990, it remains unacceptable that in 2015, one of every 20 children dies before 5 years of age. As the rates have decreased, in association with improved vaccine coverage, better health-care access, and malaria control, the remaining deaths occur disproportionately among neonates, which now account for 40–45% of childhood deaths, and in countries in parts of Africa and south Asia, where childhood mortality rates approach one in every 10 children.^1^^–^^4^ Surviving the neonatal period is becoming increasingly important to guarantee child survival, as the proportion of under-five deaths occurring in this period will continue to increase in relation to global child mortality reductions. The causes of neonatal deaths are poorly understood; thus, few interventions have been fielded against them. Verbal autopsies, a primary basis for estimating causes of death, discriminate most syndromes poorly, particularly in the neonatal period, and cannot identify causal pathogens, casting doubt over estimates used to focus, strategize, and measure progress.^5^^,^^6^ In the highest mortality regions in sub-Saharan Africa and south Asia, even verbal autopsy data are largely unavailable. For example, Global Burden of Disease estimates for cause-specific childhood mortality in the Democratic Republic of the Congo are modeled from two verbal autopsy studies published in 1989 and 1993, and one household survey based on report from the head of household (not verbal autopsy), published in 2006.^7^ Continued reliance on such data, coupled with increasing sophistication of modeling methods used, risks creating a false sense of assuredness regarding what is known about the causes of childhood mortality.

The United Nations has recently adopted ambitious Sustainable Development Goals, including the “end of preventable deaths of newborns and children under 5 years of age” by 2030.^8^ To realistically achieve such goals, the global community cannot continue to rely on uncertain, unrepresentative, and untimely mortality data. Deliberately implemented, more rigorous surveillance is needed to provide accurate information on causes of death, based on pathology specimens from children who have died. The findings must then be shared as soon as possible with families, government agencies, and global stakeholders.^6^ A novel approach, minimally invasive tissue sampling (MITS), builds on postmortem needle biopsies of tissue that have been in occasional use for disease-specific surveillance and outbreak investigations for decades. Innovative approaches have yielded rapid recognition of MITS as a comprehensive tool to obtain postmortem samples in challenging settings, demonstrating agreement with complete diagnostic autopsy in some 70–90% of cases.^9^^,^^10^ Preliminary results of a research project to validate MITS against gold standard methods in Mozambique and Brazil have shown that MITS has high agreement with, and can reliably substitute for, full autopsy, particularly for infectious causes of death.^11^ Over the past 18 months, MITS has been performed in an array of countries including Bangladesh, Brazil, Kenya, Mozambique, and South Africa, and has been acceptable to families. A multicountry anthropological research study has found good theoretical acceptability and willingness to know cause of death in the community.^12^ A recently completed hospital-based postmortem investigation in Soweto conducted among 365 stillbirths from June 2015 to September 2015, identified Group B *Streptococcus* to be definitely associated with 5% of the stillbirths and possibly related to a further 15%. This novel finding has the potential to change national policy in management and intervention in pregnant women to reduce a previously unrecognized, but potentially preventable cause of stillbirth. (S. A. Madhi, personal communication).

To address the problems of validity, timeliness, and regional representation described above, the Bill & Melinda Gates Foundation is funding the Child Health and Mortality Prevention Surveillance (CHAMPS) network. Coordinated by Emory University in association with the International Association of National Public Health Institutes, U.S. Centers for Disease Control and Prevention, and the Public Health Informatics Institute of the Task Force for Global Health, CHAMPS aims to more definitively determine the causes of childhood mortality in the highest-mortality regions of sub-Saharan Africa and south Asia, eventually including 12–15 sites. Each site will determine the causes of childhood death using MITS, in combination with conducting verbal autopsies and medical record abstractions, within demographically defined populations, producing ongoing cause-specific childhood mortality rates. Comprehensive anthropological work will be conducted to promote community engagement and sensitization, to increase the likelihood that MITS is welcomed by the communities and health-care providers, as well as religious, cultural, and political leaders.^13^ Ministry of Health and/or National Public Health Institute staff at each CHAMPS site will be engaged in implementation, analyses, and reporting to maximize the utility of evidence derived from CHAMPS for public health policy decision-making and local laboratory and pathology capacity will be built and supported to enable long-term sustainability and expansion. Protocols will be standardized across sites, and data will be uploaded to cloud-based servers for rapid dissemination to host governments and global stakeholders, such as the United Nations Children's Emergency Fund, World Health Organization, The World Bank, and disease burden expert groups. CHAMPS will seek to integrate with existing surveillance by aligning protocols and combining data with complementary systems, such as Sample Registration Systems^14^ and health management information systems to provide a current, comprehensive, and statistically valid view of childhood mortality. Six sites have been selected, and the first MITS data began flowing in January 2017.

Although we advocate MITS whenever possible for cause of death determination, we recognize that verbal autopsy and medical certification will remain more widely used because they can be done with less training and inexpensively—when MITS and verbal autopsy/medical certification are combined, they can provide more precise and statistically valid estimates of causes of death across all geographies of a country.^6^^,^^15^ Indeed, the recently funded Countrywide Mortality Surveillance for Action initiative in Mozambique will measure mortality across the country using representative sampling with verbal autopsy that is supplemented by MITS-based cause of death data from the CHAMPS site in Manhiça, as well as selected additional MITS in other parts of the country. We hope that this system can serve as a model for sample registration system solutions to civil registration and vital statistics (CRVS) in response to the global call for development and strengthening of CRVS.^16^

No child should die of a preventable cause in the new millennium. Pathology-based surveillance offers the potential for more accurately tracking the causes of death, enabling policymakers to match this information with precision public health strategies, applying policies that are specific, impactful, and responsive to changing epidemiology.^15^ We hope that CHAMPS will kindle a wide-scale demand and multifaceted financial support for strengthening public health systems to identify and tackle substantive contributors to unacceptably high death rates. However, Gates Foundation funding alone will not be enough to enable these outcomes. We encourage expanded use of surveillance linked to pathology-based cause of death determination at local, national, and global levels to provide the information needed to end preventable childhood mortality.
